# Supplementary material for: Subsurface nickel boosts the low-temperature performance of a boron oxide overlayer in propane oxidative dehydrogenation
Source: Nat Commun. 2023 Mar 17;14:1478. doi: 10.1038/s41467-023-37261-x (PMC10023692; doi:10.1038/s41467-023-37261-x)
Supplement: Supplementary file 1 — Supplementary Information [file 41467_2023_37261_MOESM1_ESM.pdf]

Supporting Information

Subsurface Nickel Boosts the Low-Temperature Performance of a Boron Oxide Overlayer in Propane Oxidative Dehydrogenation

Xiaofeng Gao [1,2†], Ling Zhu [3†], Feng Yang [4†], Lei Zhang [4], Wenhao Xu [1], Xian Zhou [1], Yongkang Huang [1], Houhong Song [1], Lili Lin [5], Xiaodong Wen [3*], Ding Ma [2*], Siyu Yao [1*]

1. Key Laboratory of Biomass Chemical Engineering of Ministry of Education, College of Chemical and Biological Engineering, Zhejiang University, Hangzhou 310027, China.
2. Beijing National Laboratory for Molecular Sciences, College of Chemistry and Molecular Engineering and College of Engineering, Peking University, Beijing 100871, China.
3. State Key Laboratory of Coal Conversion, Institute of Coal Chemistry, Chinese Academy of Sciences, Post Office Box 165, Taiyuan, Shanxi 030001, China.
4. Department of Chemistry, Guangdong Provincial Key Laboratory of Catalysis, Southern University of Science and Technology, Shenzhen 518055, China
5. Institute of Industrial Catalysis, State Key Laboratory of Green Chemistry Synthesis Technology, College of Chemical Engineering, Zhejiang University of Technology, Hangzhou 310014, Zhejiang, China

Table of Content

Experimental Section	4
Figure S1 The TEM and Ni particle distribution of BN, 4%Ni/BN and 4%Ni@BO_x/BN catalysts.	7
Figure S2 The SEM of BN, 4%Ni/BN and 4%Ni@BO_x/BN catalysts.	8
Figure S3 Microregion EELS spectra of the 4%Ni@BO_x/ BN catalyst.	9
Figure S4 Microregion EELS spectra of Ni of the 4%Ni@BO_x/ BN catalyst.	10
Figure S5 HRTEM images of Ni particle of 4% Ni@BO_x/BN catalyst.	11
Figure S6 XRD patterns of BN, 4%Ni/BN, 4%Ni@BO_x/BN, Ni/BN-W and Ni/BN-A catalysts.	12
Table S1 Texture parameters of BN, 4%Ni/BN, 4%Ni@BO_x/BN, Ni/BN-W and Ni/BN-A catalysts	13
Table S2 The surface and bulk compositions of BN,4%Ni/BN, 4%Ni@BO_x/BN, Ni/BN-W and Ni/BN-A catalysts	14
Figure S7 In-situ powder X-ray diffraction (XRD) patterns of the Ni/BN and Ni@BO_x/BN in oxygen at elevated temperature.	15
Table S3 The H_2 chemisorption results of 4%Ni/BN and 4%Ni@BO_x/BN catalysts.	16
Figure S8 In-situ DRIFTS spectra of CO adsorption over 4%Ni/BN, 4%Ni@BO_x/BN and Ni/BN-W catalysts at room temperature.	17
Figure S9 B 2*p* XPS spectra and Ni/B atomic ratio and B−O percentage of BN, 4%Ni/BN, 4%Ni@BO_x/BN, Ni/BN-W and Ni/BN-A catalysts.	18
Figure S10 ODHP reaction over 4%Ni@BO_x/BN catalyst.	19
Figure S11 The XRD pattern of NiO/BN catalyst and the catalytic activity of BN, Ni@BO_x/BN and NiO/BN catalysts.	20
Figure S12 XRD patterns of Ni@BO_x/BN catalysts with different Ni loading.	21
Figure S13 The TEM and Ni particle distribution of Ni@BO_x/BN catalysts with different Ni loading.	22
Table S4 The BET area surface and Ni surface area of BN and Ni@BO_x/BN catalysts with different Ni loading.	23
Table S5 Specific and areal rates of alkane consumption for BN and BO_x@Ni portion.	24
Figure S14 ODHP reaction over 4%Ni@BO_x/BN catalysts (WHSV =57600 $L \cdot kg^{-1} \cdot h^{-1}$).	25
Figure S15 Comparison of olefin productivity of ODPH as a function of C_3H_8 conversion for 4%Ni@BO_x/BN and several representative catalysts.	26
Figure S16 Propane consumption rate as a function particle size, propane conversion as a function of W_{cat}/F_o for various BN masses and various ID tube.	27
Figure S17 High resolution STEM images and EELS element mapping of Ni@BOx/BN-spent catalyst	28
Figure S18 XRD patterns of the fresh and spent 4%Ni@BO_x/BN catalyst.	29
Figure S19 B 2*p* XPS spectra and Ni/B atomic ratio and B−O percentage of 4%Ni@BO_x/BN4%Ni@BO_x/BN-Spent catalysts.	30
Table S6 Texture parameters of the fresh and spent 4%Ni@BO_x/BN catalyst.	31
Figure S20 Time-resolved in-situ DRIFTS observation and normalized intensity of the O-H stretching vibration.	32

Figure S21 DFT models of B_2O_3 and B_2O_3/Ni(111) after structure optimization.	33
Table S7 Bader charge analysis of the B-O and B-O-H bonds.	34
Figure S22 Spatial distribution of electron density near the B_2O_3 and Ni(111) interface.	35
Figure S23 The optimized configuration of each states in the DFT calculations.	36
Figure S24 Structure of the B_2O_3-Ni(111) interface from state D to F.	37
Table S8 The surface and gas phase conversion pathway of propane.	38

Supplementary Methods

Materials and Methods

Materials. Hexagonal boron nitride (BN) was purchased from Alfa Aisa Inc. Nickel nitrate hexahydrate ($Ni(NO_3)_2·6H_2O$) and ethanol (C_2H_5OH) were purchased from Shanghai Aladdin Bio-Chem Technology Co., Ltd. These raw materials were used as received without further treatment.

Synthesis. x%Ni/BN: x%Ni/BN was prepared by an impregnation method. In a typical synthesis process, firstly, the hexagonal boron nitride (BN) was dispersed into ethanol with the amount ratio of m_{BN}: $V_{ethanol}$ =1g:10 mL, and the suspension was sonicated for 30 minutes. Then, the required amount of nickel nitrate hexahydrate ($Ni(NO_3)_2·6H_2O$) was added to the suspension by controlling the nickel loading to x% wt. The resulting mixed solution was evaporated to powder at room temperature under strong mechanical agitation, and subsequently placed in an oven to dry overnight at 70 ℃. Finally, the samples were further treated in a pure hydrogen stream at 500 ℃ for 2 h to obtain the x%Ni/ BN catalysts, where the x was denoted as mass fraction of Ni element, and 2 wt%, 4 wt%, 6 wt% and 10 wt% were employed.

The Ni@BO_x/BN catalysts were obtained by the oxidation of x%Ni/BN samples in a 20 vol% CO_2/N_2 mixed gas flow at 800 ºC for 2 h.

The Ni/BN-W catalyst was obtained by immersing Ni@BO_x/BN catalyst into boiled water under a strong mechanical agitation for 2 h, drying overnight at 70 ℃ and heating in a pure hydrogen stream at 500 ℃ for 2 h.

The Ni/BN-A catalyst was obtained by treating Ni@BO_x/BN catalyst with 10% HNO_3 under a strong mechanical agitation for 12 h, drying overnight at 70 ℃ and heating in a pure hydrogen stream at 500 ℃ for 2 h.

Catalyst characterization. The (in-situ) powder diffraction patterns (XRD) of the Ni/BN series catalysts were collected using an XPERT-3 diffractometer with Cu Kα1 radiation (1.540598 Å) as the incident X-ray. The scan rate was set at 2°/min with a step of 0.02°. The mass specific surface areas were determined using N_2 physisorption method. The experiments were performed using a BELSORP-mini instrument. The loadings of Ni in the Ni/BN series catalysts were determined using the inductive coupling plasma atomic emission spectroscopy (ICP-AES) over a Varian ICP-OES-720 instrument. In the sample preparation, the Ni portion was dissolved from catalyst powder by aqua regia. The resulted solution was then diluted to desirable concentration for further measurement. The STEM images of the Ni/BN series catalysts were collected by a JEOL 2100 microscope equipped with EELS analyzer. The Ni/BN series catalysts were dispersed in ethanol and sonicated to make the suspension of catalysts. One drop of the suspension was transferred to the carbon grid as the sample for electron microscopy characterization. The scanning electron microscopy (SEM) investigation was carried out with a Hitachi FESEM SU8220 electron microscopes. The in-situ DRIFTS of Ni/BN series catalysts for propane oxidative dehydrogenation under reaction conditions were recorded using a Nicolet iS 20 FT-IR spectrometers equipped with an MCT-A detector, KBr windows and a HARRICK in situ cell. 0.1 g of the sample was loaded into the in situ reaction cell and was in situ pretreated in a flow (20 ml min^{-1}) of N_2 at 200 °C for 1 h and then heated to reaction temperature (440 °C). The background spectrum was collected in the continuous N_2 flowing. A mixture gas of 16.7vol% C_3H_8 + 25vol% O_2 + 58.3vol% N_2 (5 ml min^{-1}) was subsequently introduced into the reaction cell, and the spectra were collected for 4 h. After that, the mixture gas was instantaneously switched to 25vol% O_2 + 75vol% N_2 (5 ml min^{-1}) and the

spectra were collected for another 2 h. Finally, the mixture gas was instantaneously switched back to 16.7vol% C_3H_8 + 25vol% O_2 + 58.3vol% N_2 (5 ml min^{-1}) and the spectra were collected for 3 h. The DRIFT spectra were obtained in the range of 4000 to 650 cm^{-1} with a resolution of 4 cm^{-1} and 64 scans. The CO probe DRIFTS of the 4% Ni/BN, 4% Ni@BO_x/BN and Ni/BN-W catalysts were collected using a Bruker Vortex 80 spectrometer equipped with diffused reflectance accessories. The fine powders of the catalysts were loaded in the cell and the background spectrum was collected at room temperature in Ar flow. Then, the sample was exposed to 20% CO in Ar until reaching steady state. The cell was purged with Ar flow to remove physiosorbed CO and the IR spectra were collected in the meantime until no change occurred. The last spectrum was used for analysis. The (In-situ) X-ray photoelectron spectra (XPS) analysis was performed using a Thermo Scienti fic ESCALAB 250Xi spectrometer using an Al K α X-ray source and pass energy of 20 eV. The C 1s peak located at 284.5 eV was used to calibrate binding energy positions. H_2 Pulse chemisorption was recorded using an AutoChemII 2920 station from Micromeritics. 100 mg x% Ni/BN-W catalysts were loaded into U-shaped quartz reactor with an inner diameter of 0.5 cm. Before the test, the sample was pretreated in a flow (30 ml min^{-1}) of H_2 at 500 °C for 1 h and then maintained 400 °C for another 30 min with switching to an argon flow of 30 mL/min. After that, the sample was cooled to 30 °C for chemisorption in order to clean the Ni surface and to avoid the presence of residual adsorbed hydrogen. The H_2 pulse chemisorption was performed at 30 °C and the volume of the injection loop was 0.5 cm^3. The carrier gas was Ar in the case of H_2 pulses. The H_2 consumptions were measured by a thermal conductive detector (TCD).

Catalytic performance test. Taking 4%Ni@BO_x/BN catalyst as an example, in a typical experiment, 200 mg of 4%Ni@BO_x /BN catalyst was loaded in a fixed bed tube furnace reactor with an inner diameter of 8 mm. The reactant gas consists of 16.7vol% C_3H_8, 25vol% O_2 and 58.3vol% N_2, and a reactant flow rate of 24 mL/min was employed with a weight hourly space velocity (WHSV) of 7200 mL/($g_{cat.}$·h). The sample was heated to the designated reaction temperature (400–500 °C) in the flow of reactant gas. The products of the reaction were analyzed by an on-line gas chromatography (GC, Agilent 8890) equipped with a thermal conductive detector (TCD) and a flammable ionization detector (FID). The N_2 in the flow was used as the inner standard. The response factors of reactants and products were calibrated using standard curve methods.

Equations

The conversion of the C_3H_8 is defined as:

$$\mathrm{Conv.}(\mathrm{C_3H_8}) = (F(\mathrm{C_3H_8})_{\mathrm{inlet}} - F(\mathrm{C_3H_8})_{\mathrm{outlet}})/\mathrm{F}(\mathrm{C_3H_8})_{\mathrm{inlet}} \qquad \text{Eq (1)}$$

The selectivity of product is defined as:

$$\mathrm{Sel.}(\mathrm{prod.}) = F(\mathrm{carbon\ prod.},)_{\mathrm{outlet}}/ \left(F(\mathrm{C_3H_8})_{\mathrm{in}let} - F(\mathrm{C_3H_8})_{\mathrm{outlet}}\right) \qquad \text{Eq (2)}$$

The weight-hour-space-velocity (WHSV, mL·$g^{-1}h^{-1}$), is defined as:

$$\mathrm{WHSV} = F_{\mathrm{total}} * \left({}^{V}/_{n}\right)_{\mathrm{STP}} * 1000 * 60 / m_{\mathrm{catal.}} \qquad \text{Eq (3)}$$

F is the formation rate of certain reactants or products in the unit of mol/min. $\left({}^{V}/_{n}\right)_{\mathrm{STP}}$is the 22.5 (L/mol) at 298.15 K. $m_{catal.}$ is the mass of catalyst loaded in the reactor (g).

Details of the DFT calculations

The adsorption energy calculations were performed with the plane wave based pseudo-potential

code in Vienna *ab initio* simulation package (VASP).[1,2] The electron-ion interaction is described with the projector augmented wave method (PAW).[3,4] The exchange and correction energies are described by the generalized gradient approximation using Perdew-Burke-Ernzerhof formulation (GGA-PBE).[5] Since van der Walls dispersion interaction plays an important role in weak interaction system,[6,7] the D3 correction of Grimme was used.[8] The plane wave cutoff energy was specified by 400 eV, the electron smearing method with $\sigma = 0.20$ eV was used to ensure energies with errors due to smearing of less than 1 meV per unit cell. The convergence criteria for geometry optimizations of total energy and forces were 10^{-5} eV and 0.02 eV/Å. Spin polarization was included. The 4×4×1 Monkhorst-Pack k-points sampling was used. To avoid interactions among slabs, the vacuum layer between periodically repeated slabs was set as 15 Å. Transition state was located using the climbing image nudged elastic band (CI-NEB) method.[9]

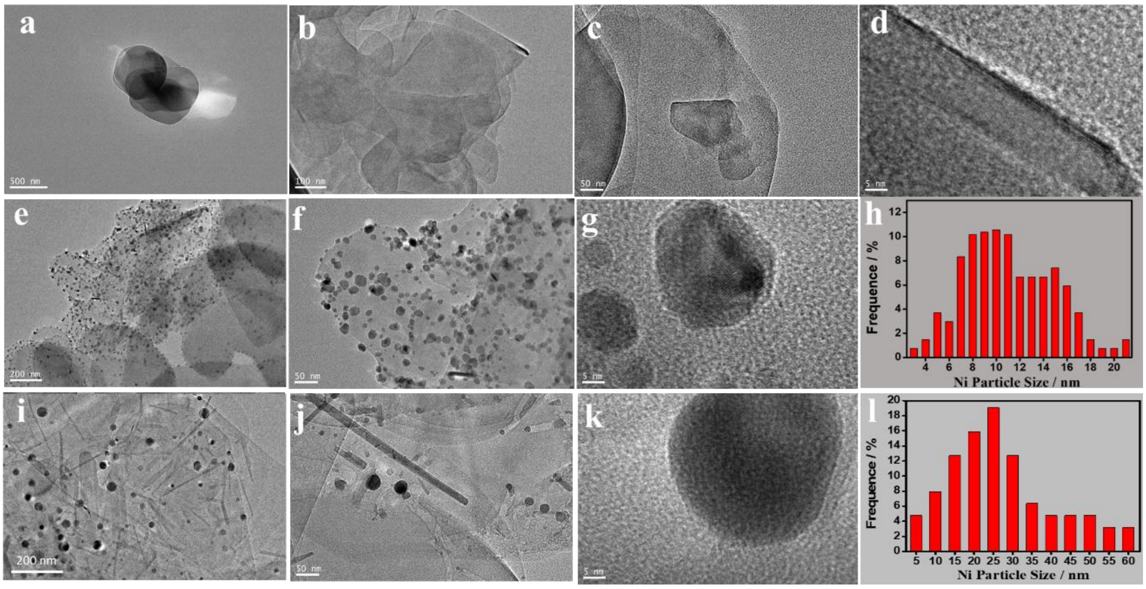

Figure S1. The TEM and Ni particle distribution of different catalysts. **a-d,** BN. **e-h**, 4%Ni/BN. **i-l**, 4%Ni@BO_x/BN.

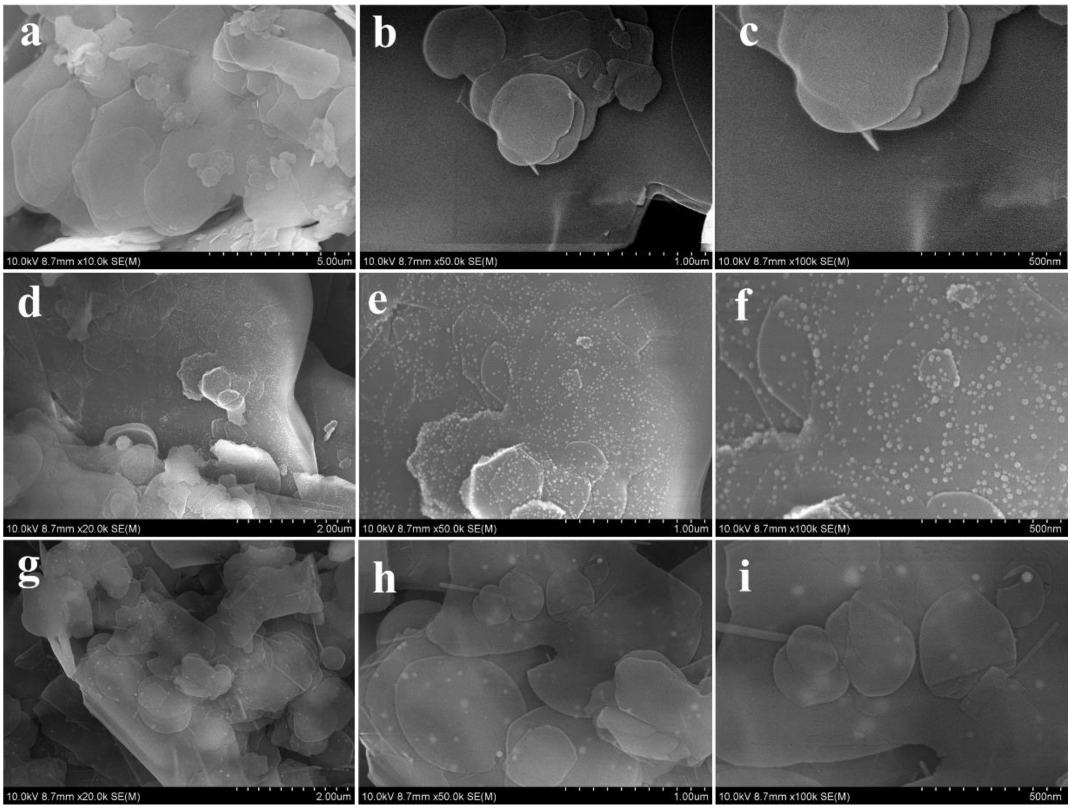

Figure S2. The SEM of different catalysts. **a-c**, BN. **d-f**, 4%Ni/BN. **g-i,** 4%Ni@BO_x/BN.

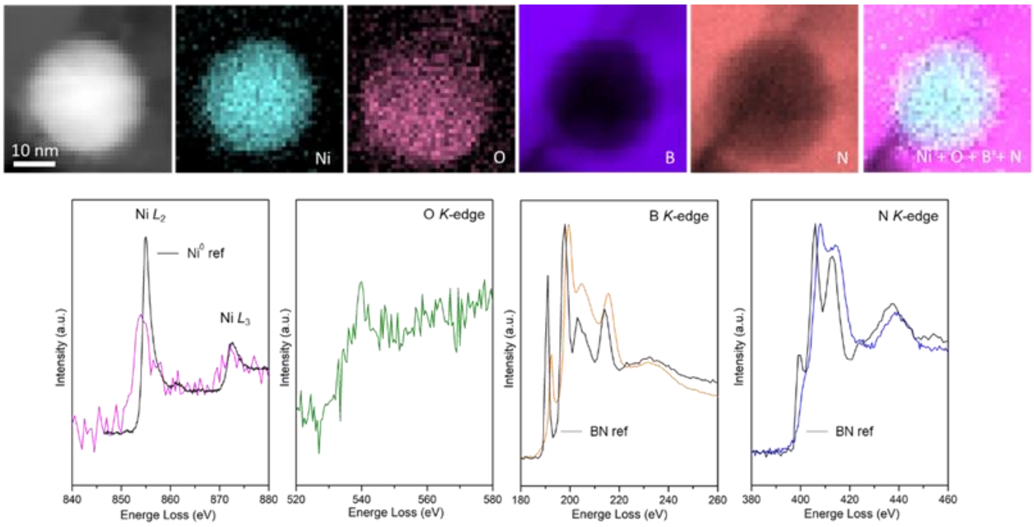

Figure S3. Microregion EELS spectra of the 4%Ni@BO_x/ BN catalyst

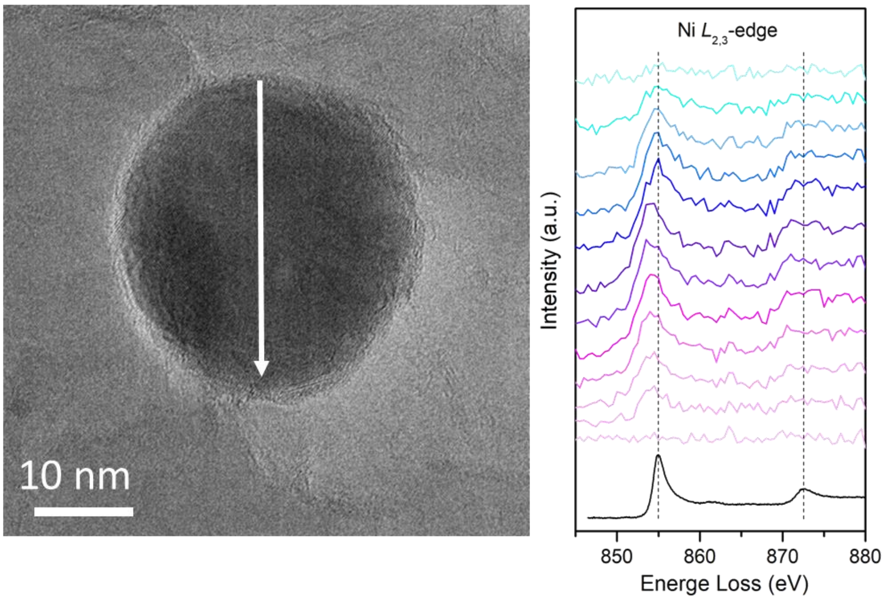

Figure S4. Microregion EELS spectra of Ni of the 4%Ni@BO_x/ BN catalyst

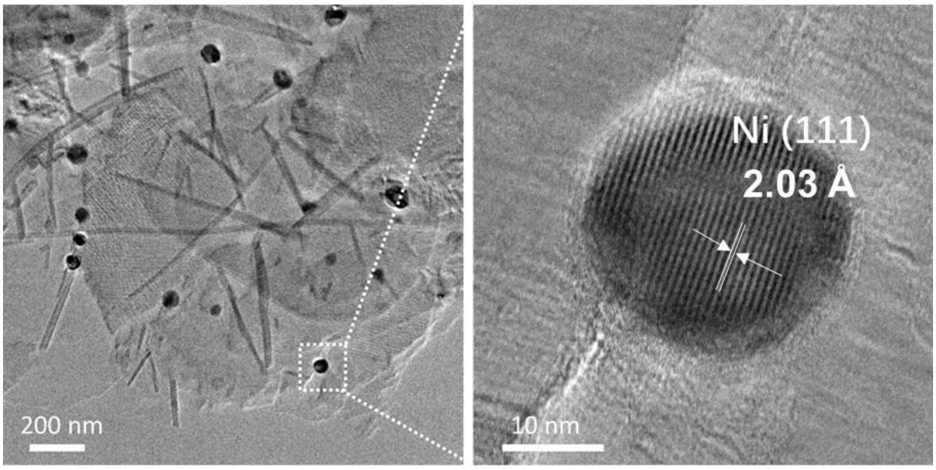

Figure S5. HRTEM images of Ni particle of 4% Ni@BO_x/BN catalyst

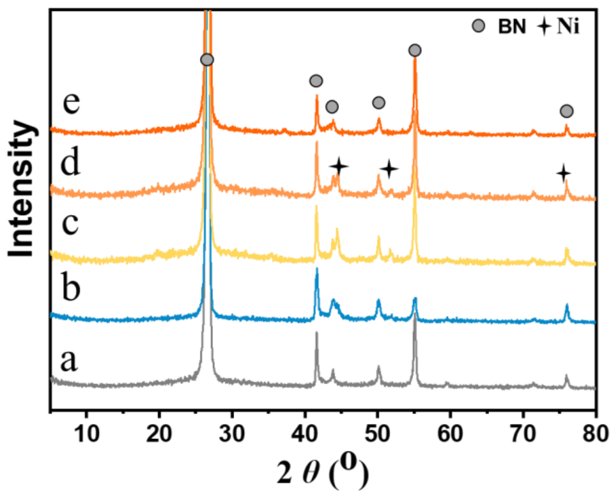

Figure S6. XRD patterns of different catalysts. **a**, BN. **b**, 4%Ni/BN. **c**, 4%Ni@BO_x/BN. **d**, Ni/BN-W. **e**, Ni/BN-A.

Table S1. Texture parameters of different catalysts

Catalysts	[a] BET area (m^2g^{-1})	[b] Pore volume (cm^3g^{-1})	[c] Average pore diameter (nm)
BN	13.075	0.065052	17.657
4%Ni/BN	16.385	0.076544	16.499
4%Ni@BO_x/BN	11.235	0.062232	20.639
Ni/BN-W	15.651	0.080649	18.675
Ni/BN-A	14.367	0.077069	18.768

[a] Specific surface area was calculated by applying the BET method.

[b] V_{Pore} was calculated from the adsorption branch at $p/p^0 = 0.99$.

[c] Average pore diameter was calculated from the BJH adsorption branch.

Table S2. The surface and bulk compositions of different catalysts

Catalysts	[a] Element Information of Catalyst Surface (atm. %)				[b] Bulk Content (wt %)
	B	N	O	Ni	Ni
BN	50.86	47.78	1.37	--	--
4%Ni/BN	53.17	43.29	2.63	0.89	4.05
4%Ni@BO_x/BN	53.11	42.71	3.75	0.42	4.55
Ni/BN-W	52.96	44.61	1.63	0.76	4.31
Ni/BN-A	51.89	46.99	1.49	0.06	0.18

[a] Surface contents by XPS analysis; [b] Bulk contents by ICP analysis.

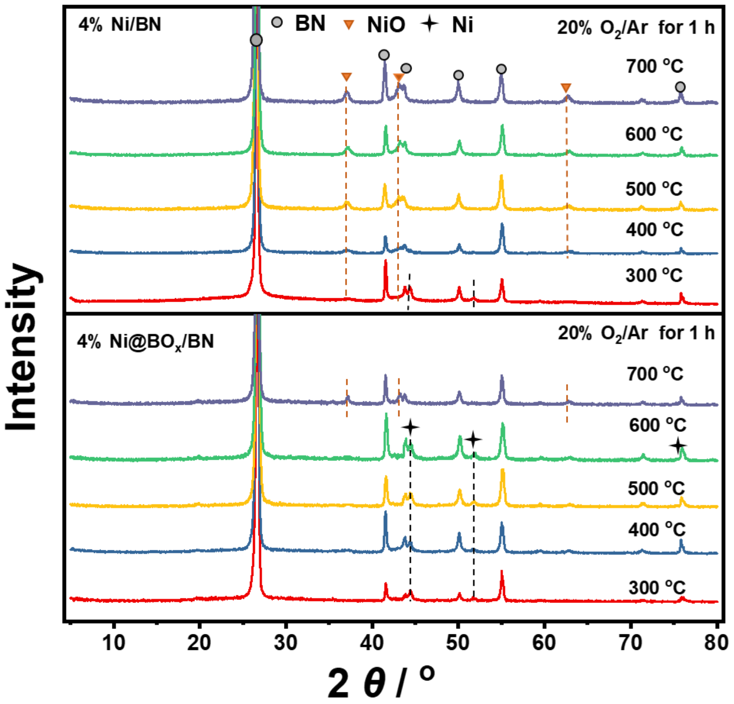

Figure S7. In-situ powder X-ray diffraction (XRD) patterns of the Ni/BN and Ni@BO_x/BN in oxygen at elevated temperature

Table S3. The H_2 chemisorption results of 4%Ni/BN and 4%Ni@BO_x/BN catalysts

Catalysts	Ni surface area (m^2g^{-1})
4%Ni/BN	0.51521
4% Ni@BO_x/BN	< 0.0001
Ni/BN-W	0.30306

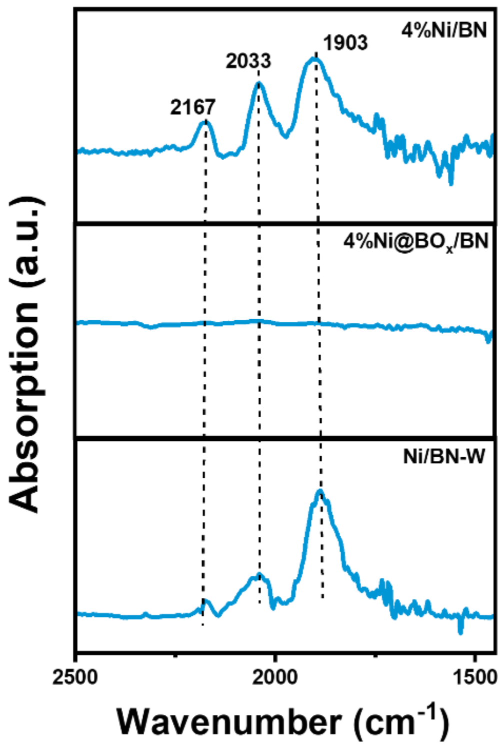

Figure S8. In-situ DRIFTS spectra of CO adsorption over 4%Ni/BN, 4%Ni@BO_x/BN and Ni/BN-W catalysts at room temperature.

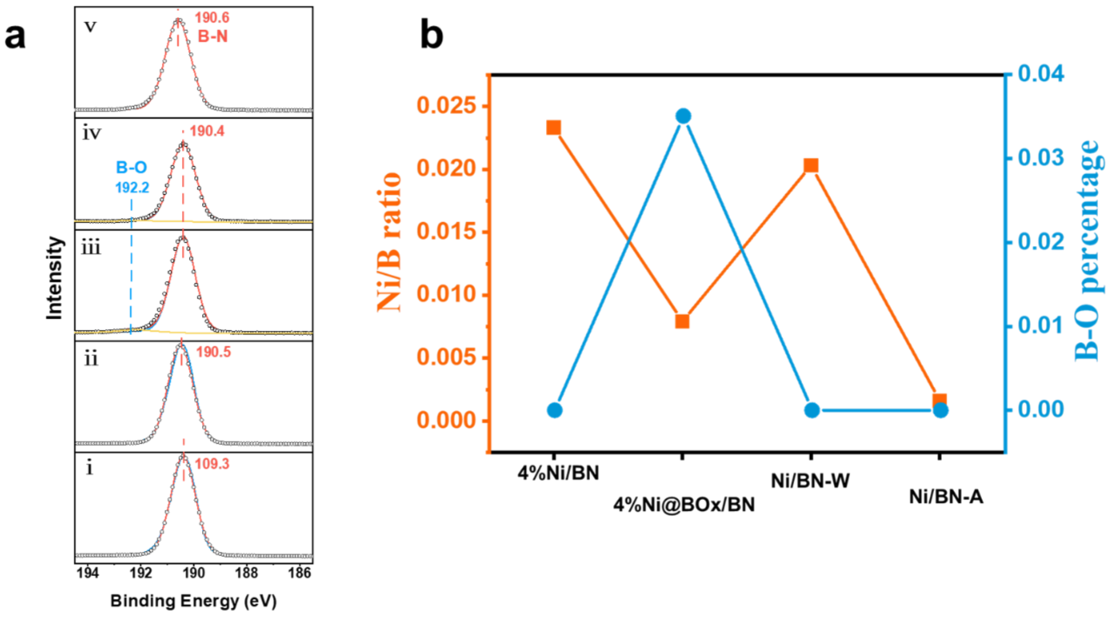

Figure S9. **a**, B 2*p* XPS spectra of different catalysts, (i) BN, (ii) 4%Ni/BN, (iii) 4%Ni@BO_x/BN, (iv) Ni/BN-W, (v) Ni/BN-A. **b**, XPS Ni/B atomic ratio and B−O percentage of the surface of different catalysts

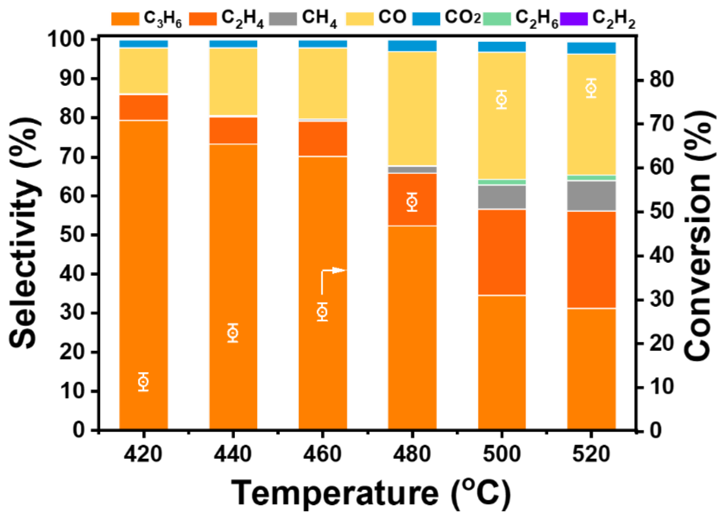

Figure S10. ODHP reaction over 4%Ni@BO_x/BN catalyst: C_3H_8 conversions and product selectivity at 420-520 °C, 0.1 Mpa, $C_3H_8/O_2/N_2$=1/1.5/3.5, WHSV =7200 L·kg^{-1}·h^{-1}

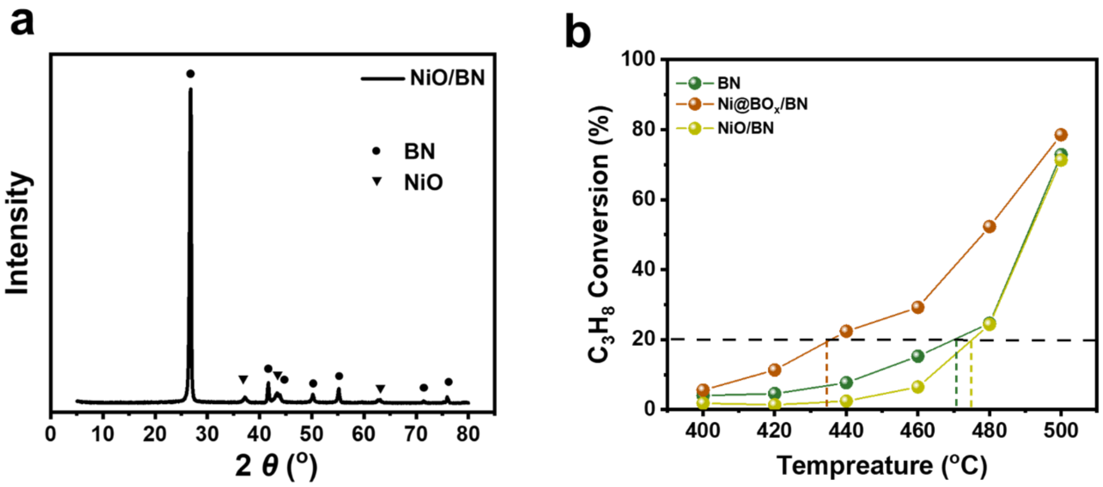

Figure S11. **a,** The XRD pattern of NiO/BN catalyst. **b**, The catalytic activity of BN, Ni@BO_x/BN and NiO/BN catalysts: 440 °C, 0.1 Mpa, $C_3H_8/O_2/N_2$=1/1.5/3.5, WHSV =7200 $L{\cdot}kg^{-1}{\cdot}h^{-1}$

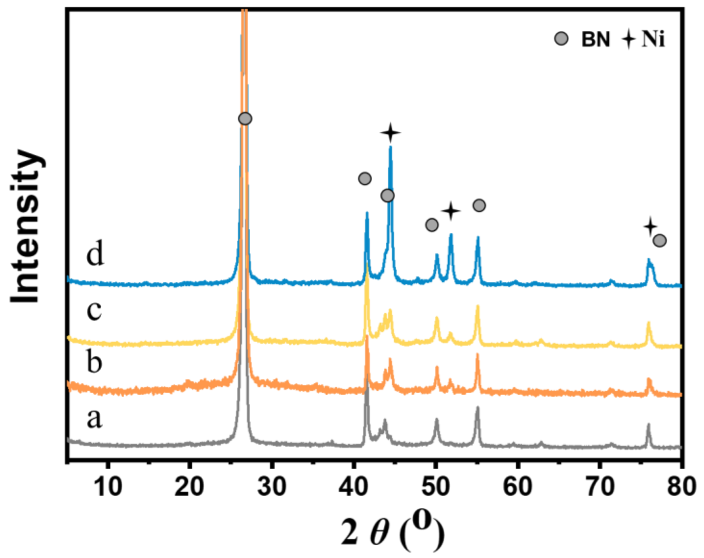

Figure S12. XRD patterns of Ni@BO_x/BN catalysts with different Ni loading. **a**, 2% Ni@BO_x/BN. **b**, 4% Ni@BO_x/BN. **c**, 6% Ni@BO_x/BN. **d**, 10% Ni@BO_x/BN.

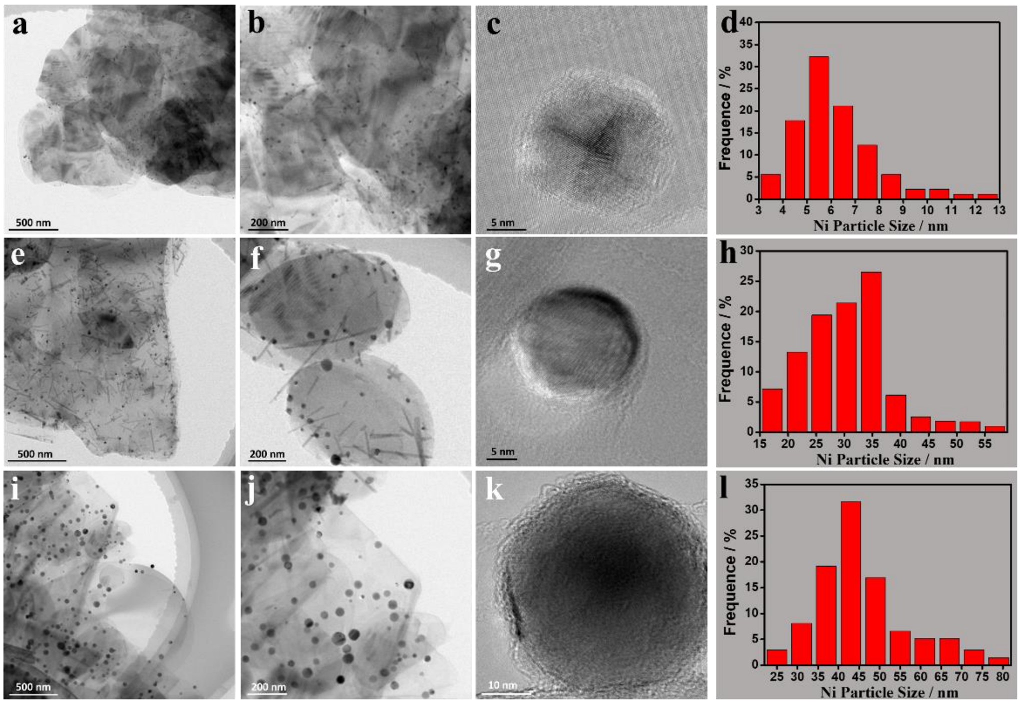

Figure S13. The TEM and Ni particle distribution of different catalysts. **a-d**, 2% Ni@BO_x/BN. **e-h**, 6% Ni@BO_x/BN. **i-l**, 10% Ni@BO_x/BN.

Table S4. The BET area surface and Ni surface area of different catalysts

Catalysts	[a] BET area (m^2g^{-1})	[b] Ni surface area (m^2g^{-1})
BN	13.075	------
2%Ni@BO_x/BN-W	13.606	0.20923
4%Ni@BO_x/BN-W	11.235	0.30306
6% Ni@BO_x/BN-W	13.784	0.01359
10% Ni@BO_x/BN-W	13.193	0.00612

[a] BET area by N_2 physisorption analysis; [b] Ni surface area by H_2 pulse chemisorption analysis.

Note: Before the H_2 pulse chemisorption analysis, all the catalysts were treated by boiled water for 1 h.

Table S5. Specific and areal rates of alkane consumption for BN and BO_x@Ni portion

Catalysts	Conversion%	Areal rate (*10^{-8})[mol $_{C3H8}$ $m^{-2}s^{-1}$]	Rate (*10^{-3})[mol $_{C3H8}$ $kg_{catal.}^{-1}s^{-1}$]
BN	7.68	8.744	------
Ni@B_2O_3 (2%Ni@BO_x/BN)	19.81	849.3	88.85
Ni@B_2O_3 (4%Ni@BO_x/BN)	22.90	809.3	61.31
Ni@B_2O_3 (6%Ni@BO_x/BN)	8.81	785.6	1.780
Ni@B_2O_3 (8%Ni@BO_x/BN)	8.07	794.9	0.486

Note: The areal or mass rate of Ni@B_2O_3 was calculated by propane molecules converted on Ni@B_2O_3 dividing the Ni surface area (obtained from Table S4) or Ni mass.

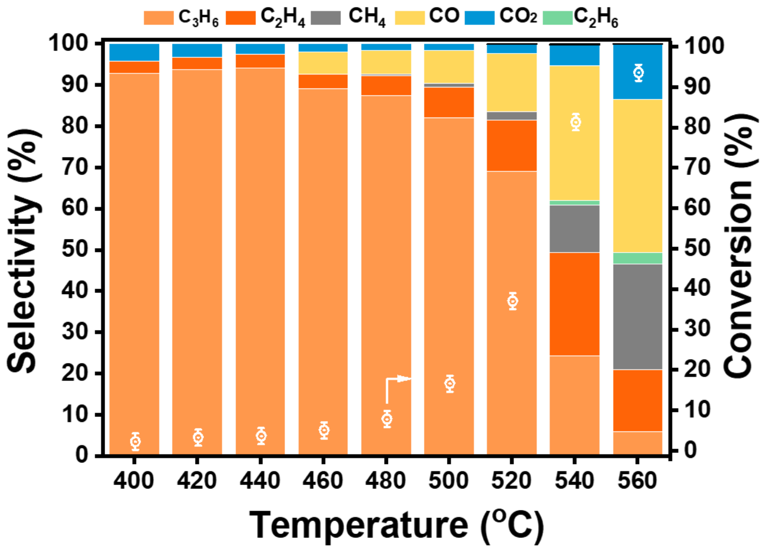

Figure S14. ODHP reaction over 4%Ni@BO_x/BN catalysts: C_3H_8 conversions and product selectivity at 400-560 °C, 0.1 Mpa, $C_3H_8/O_2/N_2$=1/1.5/3.5, WHSV =57600 L·kg^{-1}·h^{-1}

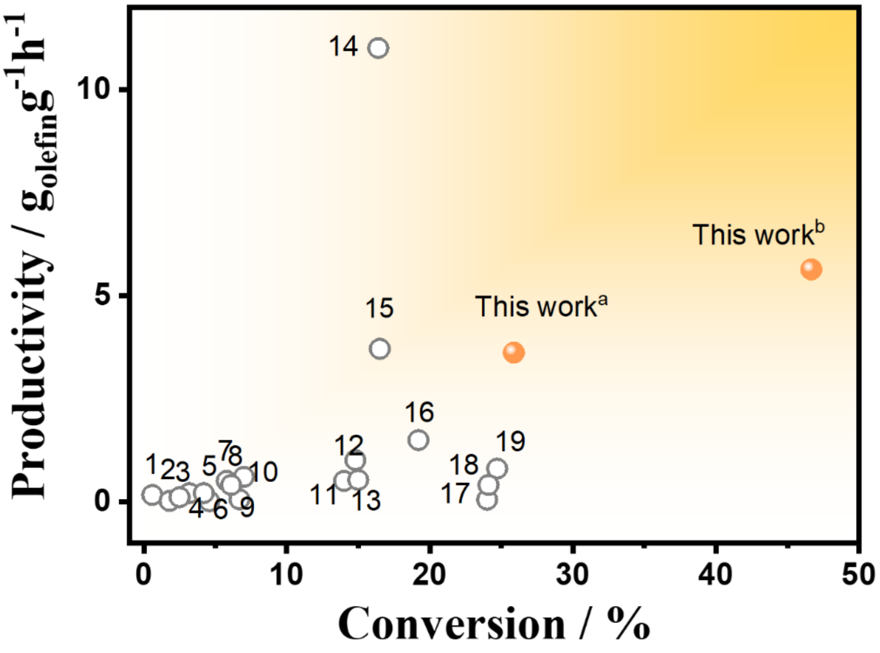

Figure S15. Comparison of olefin (C_3H_8+C_2H_4) productivity of ODPH as a function of C_3H_8 conversion for 4%Ni@BO_x/BN and several representative catalysts: This work[a] 4% Ni@BO_x/BN (500°C, 0.1 Mpa, $C_3H_8/O_2/N_2$=1/1.5/3.5, WHSV =57600 L·kg^{-1}·h^{-1}); This work[b] 4% Ni@BO_x/BN (520°C, 0.1 Mpa, $C_3H_8/O_2/N_2$=1/1.5/3.5, WHSV =57600 L·kg^{-1}·h^{-1});1 B-CNFs[10]; 2 B-Annealed ND[11]; 3 WB[12]; 4 Co_2B/Co_3B[12]; 5 HfB_2[12]; 6 B_2O_3-OCNTs[10]; 7 Ti_2B[12]; 8 NiB[12]; 9 Mesoporous B_xCN[13]; 10 B_4C[12]; 11 *h*-BN[14]; 12 B_2O_3/SBA-15[15]; 13 B-MWW[16]; 14 B[12]; 15 BNNTs[14]; 16 SiB_6[17]; 17 SS-BNNSs[18]; 18 High surface area BN[19]; 19 B_2O_3@BPO_4-800[20]

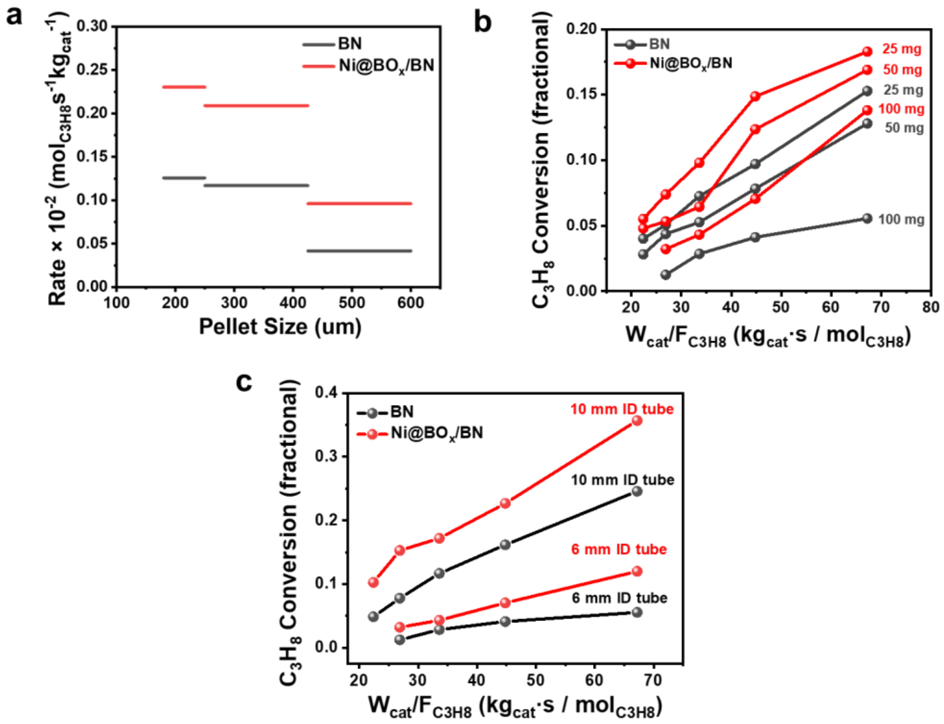

Figure S16. a, Propane consumption rate as a function particle size (reaction conditions: 440 ^{o}C, V/V/V of $C_3H_8/O_2/N_2$ = 1/1.5/3.5, 0.1 Mpa). **b**, Propane conversion as a function of W_{cat}/F_o for various BN masses (reaction conditions: 440 ^{o}C, 0.1 Mpa, WHSV =14400 L·kg^{-1}·h^{-1}). **c,** Propane conversion as a function of W_{cat}/F_o for various ID tube (reaction conditions: 440 ^{o}C, 0.1 Mpa, WHSV =14400 L·kg^{-1}·h^{-1})

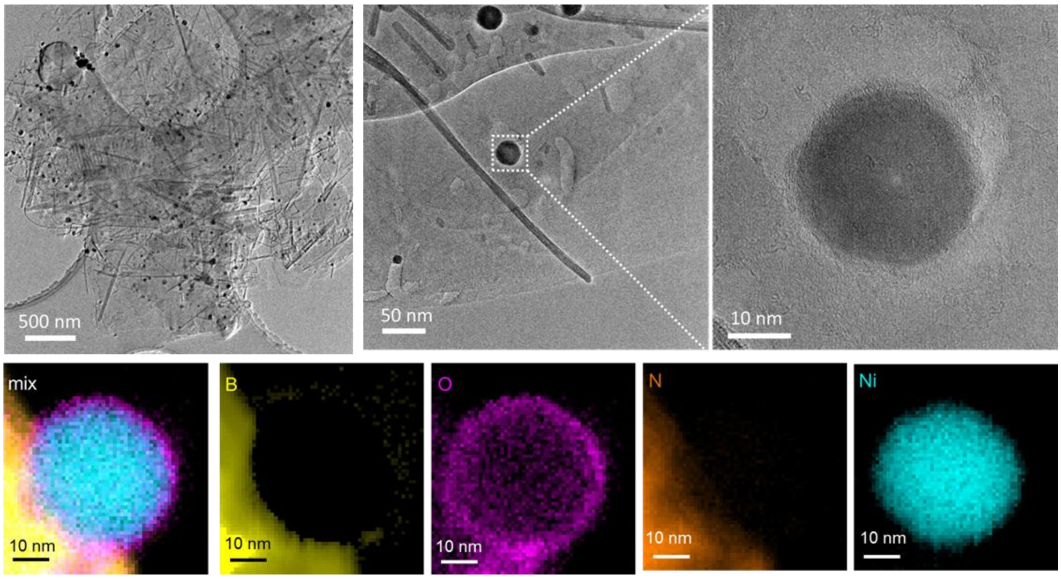

Figure S17. High resolution STEM images and EELS element mapping of Ni@BOx/BN-spent catalyst

-

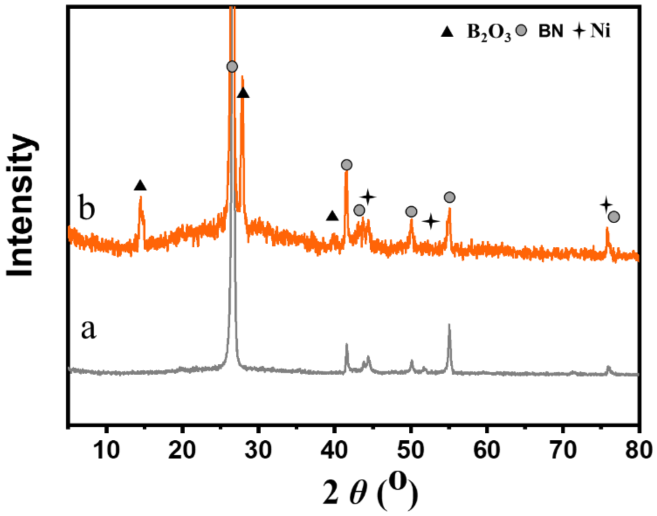

Figure S18. XRD patterns of the fresh and spent 4%Ni@BO_x/BN catalyst. **a**, 4%Ni@BO_x/BN. **b**, 4%Ni@BO_x/BN-spent.

Note: The 4%Ni@BO_x/BN-spent is the 4%Ni@BO_x/BN catalyst after 50 h ODHP reaction.

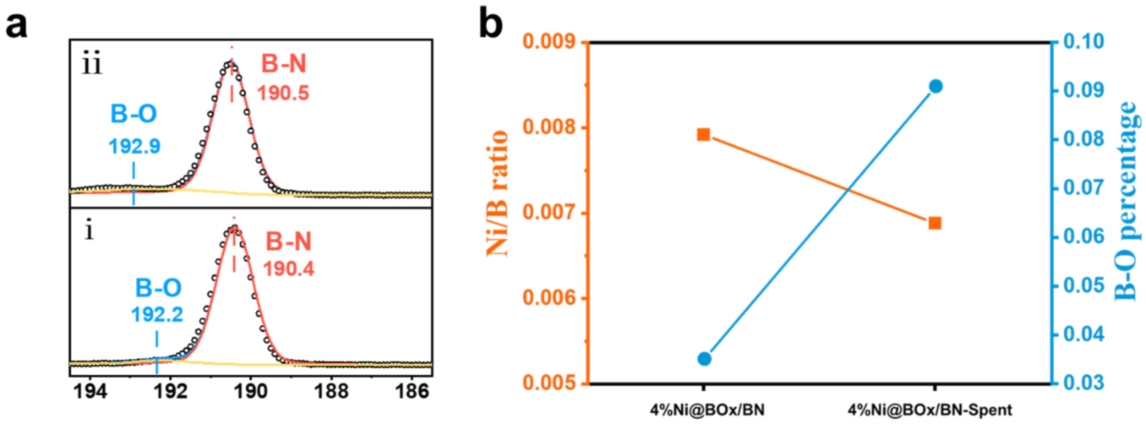

Figure S19. a, B 2*p* XPS spectra of different catalysts, (i) 4%Ni@BO_x/BN, (ii) 4%Ni@BO_x/BN-Spent. **b,** XPS Ni/B atomic ratio and B−O percentage of the surface of two catalysts

Table S6. Texture parameters of the fresh and spent 4%Ni@BO_x/BN catalyst

Catalysts	[a] BET area (m^2g^{-1})	[b] Pore volume (cm^3g^{-1})	[c] Average pore diameter (nm)
4%Ni@BO_x/BN	11.235	0.062232	20.639
4%Ni@BO_x/BN-Spent	7.7374	0.059651	34.598

[a] Specific surface area was calculated by applying the BET method.

[b] V_{Pore} was calculated from the adsorption branch at $p/p^0 = 0.99$.

[c] Average pore diameter was calculated from the BJH adsorption branch.

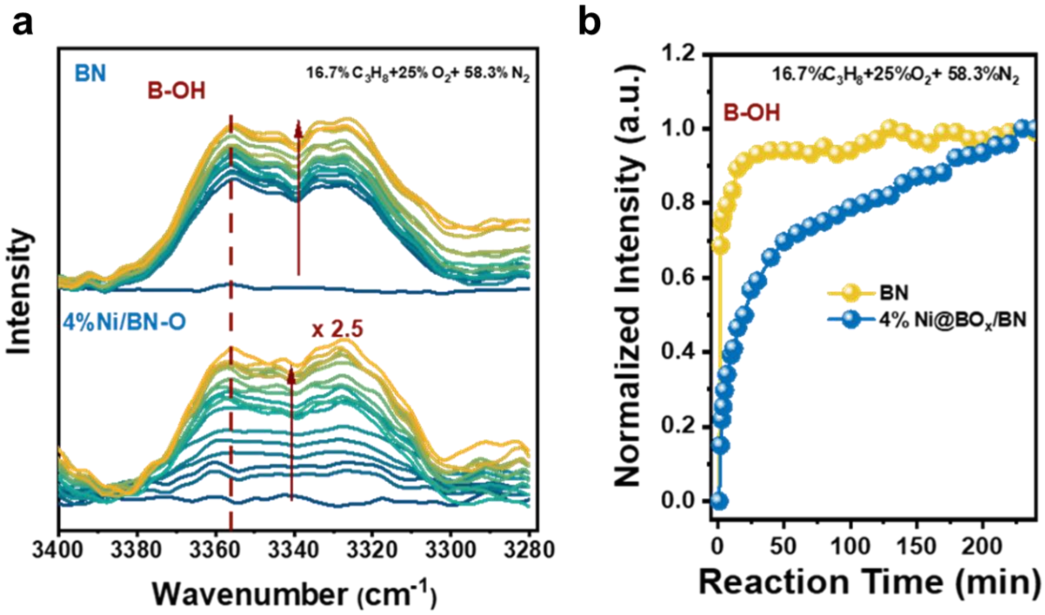

Figure S20. a, Time-resolved in-situ DRIFTS observation of the B-OH species from the beginning of the reaction to the steady state. **b**, Normalized intensity of the O-H stretching vibration peak with time from the beginning of the reaction to the steady state.

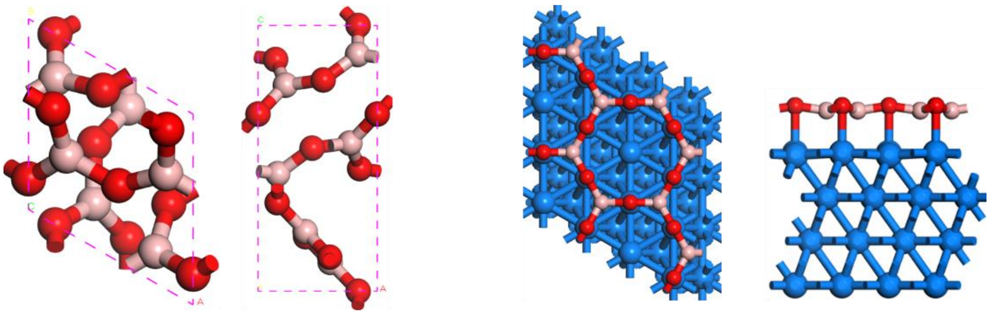

B_2O_3
$d_{(B\text{-}O)}$= 1.37 Å

B_2O_3/Ni(111)
$d_{(B\text{-}O)}$= 1.45 Å

Figure S21. DFT models of B_2O_3 and B_2O_3/Ni(111) after structure optimization.

Table S7. Bader charge analysis of the B-O and B-O-H bonds

	B	O	H
B_2O_3 (101)	+2.3	-1.5	-
B_2O_3/Ni(111)	+2.1	-1.5	-
B-O-H of B_2O_3 (101)	+2.3	-1.5	+0.8
B-O-H of B_2O_3/Ni(111)	+2.2	-1.4	+0.7

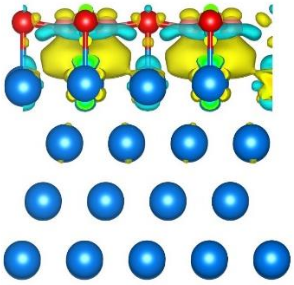

Figure S22. Spatial distribution of electron density near the B_2O_3 and Ni(111) interface.

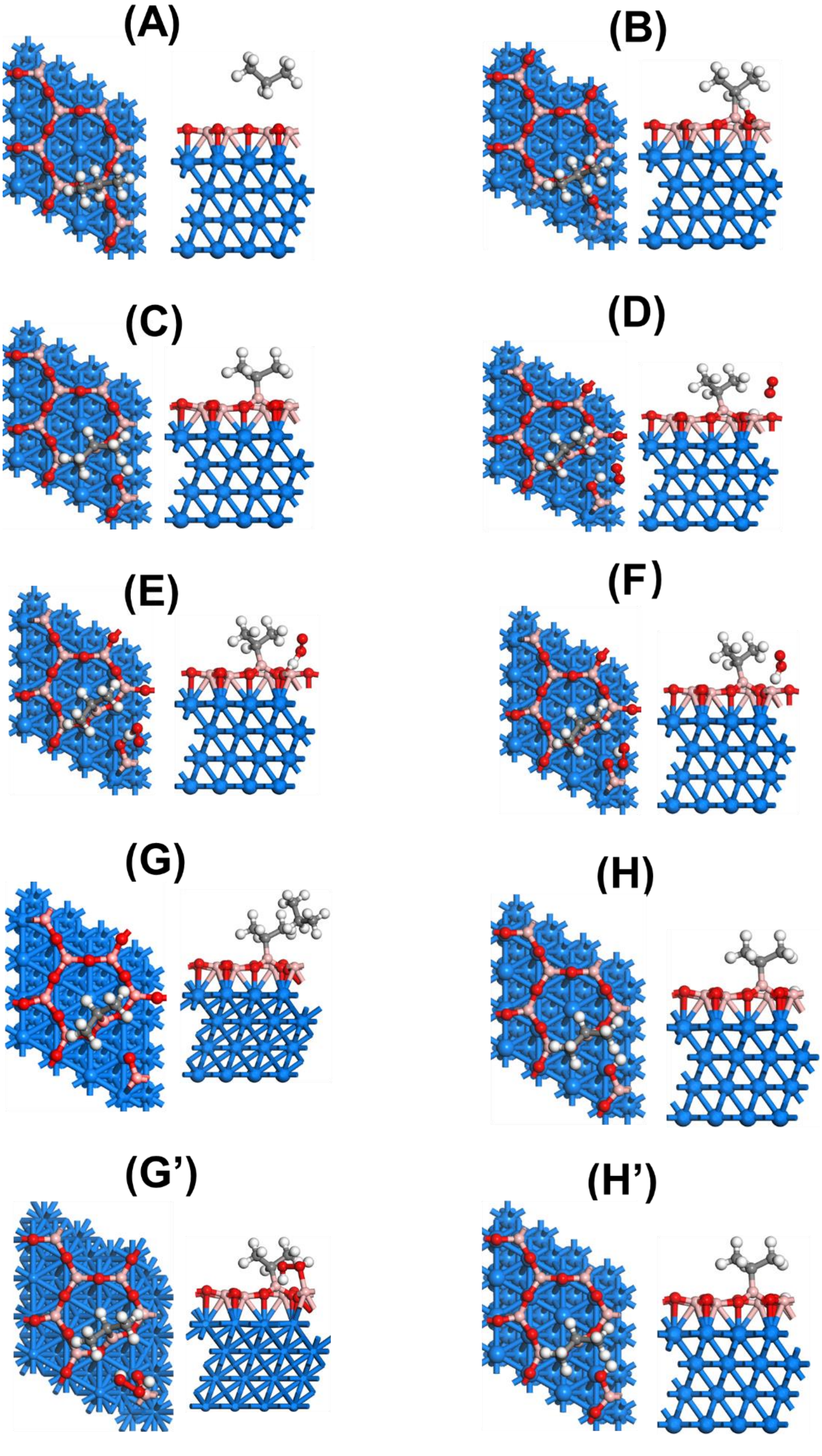

Figure S23. The optimized configuration of each states in the DFT calculations (A-H(H') corresponds to the optimized atomic configuration of each states in Figure 4 in article).

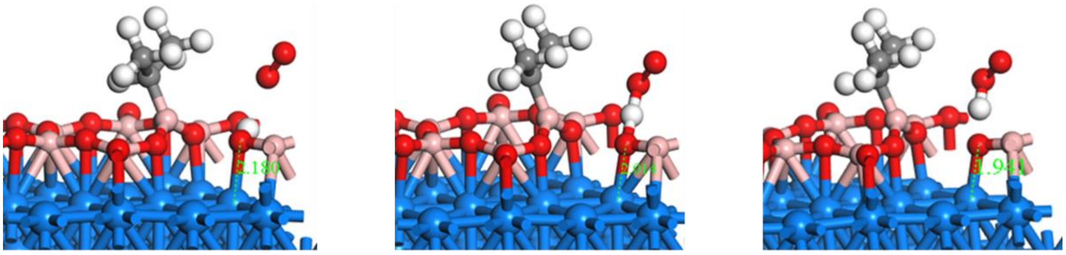

State D $d_{(Ni\text{-}O)}$= 2.180 Å

State E (TS2) $d_{(Ni\text{-}O)}$= 2.014 Å

State F $d_{(Ni\text{-}O)}$= 1.941 Å

Figure S24. Structure of the B_2O_3-Ni(111) interface from state D to F.

Table S8. The surface and gas phase conversion pathway of propane

**a: Surface conversion pathway of propane**	**b: Gas phase conversion pathway of propane**
$O_2(g) + BO\text{-}H(s) \rightarrow \cdot OOH(g) + BO\cdot(s)$<br>$BO\cdot(s) + C_3H_8(g) \rightarrow BO\text{-}H(s) + \cdot C_3H_7(g)$<br>$\cdot C_3H_7(g) + \cdot OOH(g) \rightarrow H_2O_2(g) + C_3H_6(g)$<br>$H_2O_2(g) \rightarrow H_2O(g) + 1/2\ O_2(g)$	$O_2(g) + BO\text{-}H(s) \rightarrow \cdot OOH(g) + BO\cdot(s)$<br>$\cdot OOH(g) + C_3H_8(g) \rightarrow H_2O_2(g) + \cdot C_3H_7(g)$<br>$H_2O_2(g) + BO\cdot(s) \rightarrow BO\text{-}H(s) + \cdot OOH(g)$<br>$\cdot C_3H_7(g) + \cdot OOH(g) \rightarrow H_2O_2(g) + C_3H_6(g)$<br>$H_2O_2(g) \rightarrow H_2O(g) + 1/2\ O_2(g)$
**Sum:**<br>$C_3H_8(g) + 1/2\ O_2(g) \rightarrow C_3H_6(g) + H_2O(g)$	**Sum:**<br>$C_3H_8(g) + 1/2\ O_2(g) \rightarrow C_3H_6(g) + H_2O(g)$

Note: a, The surface conversion pathway of propane. **b**, The gas phase conversion pathway of propane. In the former pathway, BO· reacts with a propane molecule to finish the H abstract reaction; While in the latter pathway, the BO· reacts with a H_2O_2 intermediate to indirectly grasp H from a propane molecule.[21,22] To simplify the process, the adsorption and desorption steps are not shown in the chemical equations.

Supplementary References

1 Kresse, G. & Furthmüller, J. Efficiency of ab-initio total energy calculations for metals and semiconductors using a plane-wave basis set. *Comput. Mater. Sci.* **6**, 15-50 (1996).

2 Kresse, G. & Furthmüller, J. Efficient iterative schemes for ab initio total-energy calculations using a plane-wave basis set. *Phys. Rev. B* **54**, 11169-11186 (1996).

3 Blöchl, P. E. Projector augmented-wave method. *Phys. Rev. B* **50**, 17953-17979 (1994).

4 Kresse, G. & Joubert, D. From ultrasoft pseudopotentials to the projector augmented-wave method. *Phys. Rev. B* **59**, 1758-1775 (1999).

5 Perdew, J. P., Burke, K. & Ernzerhof, M. Generalized Gradient Approximation Made Simple. *Phys. Rev. Lett.* **77**, 3865-3868 (1996).

6 Silva, J. L. F. D., Stampfl, C. & Scheffler, M. Adsorption of Xe Atoms on Metal Surfaces: New Insights from First-Principles Calculations. *Phys. Rev. Lett.* **90**, 066104 (2003).

7 Da Silva, J. L. F., Stampfl, C. & Scheffler, M. Xe adsorption on metal surfaces: First-principles investigations. *Phys. Rev. B* **72**, 075424 (2005).

8 Grimme, S., Antony, J., Ehrlich, S. & Krieg, H. A consistent and accurate ab initio parametrization of density functional dispersion correction (DFT-D) for the 94 elements H-Pu. *J. Chem. Phys.* **132**, 154104 (2010).

9 Henkelman, G., Uberuaga, B. P. & Jónsson, H. A climbing image nudged elastic band method for finding saddle points and minimum energy paths. *J. Chem. Phys.* **113**, 9901-9904 (2000).

10 Sheng, J. *et al.* Oxidative dehydrogenation of light alkanes to olefins on metal-free catalysts. *Chem. Soc. Rev.* **50**, 1438-1468 (2021).

11 Sun, X., Ding, Y., Zhang, B., Huang, R. & Su, D. S. New insights into the oxidative dehydrogenation of propane on borate-modified nanodiamond. *Chem. Commun.* **51**, 9145-9148 (2015).

12 Grant, J. T. *et al.* Boron and Boron-Containing Catalysts for the Oxidative Dehydrogenation of Propane. *ChemCatChem* **9**, 3623-3626 (2017).

13 Goyal, R. *et al.* Single-step synthesis of hierarchical B_xCN: a metal-free catalyst for low-temperature oxidative dehydrogenation of propane. *J. Mater. Chem. A* **4**, 18559-18569 (2016).

14 Grant, J. T. *et al.* Selective oxidative dehydrogenation of propane to propene using boron nitride catalysts. *Science* **354**, 1570-1573 (2016).

15 Lu, W.-D. *et al.* Supported Boron Oxide Catalysts for Selective and Low-Temperature Oxidative Dehydrogenation of Propane. *ACS Catal.* **9**, 8263-8270 (2019).

16 Qiu, B. *et al.* Oxidative dehydrogenation of propane using layered borosilicate zeolite as the active and selective catalyst. *J. Catal.* **385**, 176-182 (2020).

17 Yan, B., Li, W.-C. & Lu, A.-H. Metal-free silicon boride catalyst for oxidative dehydrogenation of light alkanes to olefins with high selectivity and stability. *J. Catal.* **369**, 296-301 (2019).

18 Cao, L. *et al.* Spherical Superstructure of Boron Nitride Nanosheets Derived from Boron-Containing Metal–Organic Frameworks. *J. Am. Chem. Soc.* **142**, 8755-8762 (2020).

19 Chaturbedy, P., Ahamed, M. & Eswaramoorthy, M. Oxidative Dehydrogenation of Propane over a High Surface Area Boron Nitride Catalyst: Exceptional Selectivity for Olefins at High Conversion. *ACS Omega* **3**, 369-374 (2018).

20 Liu, Q. *et al.* B_2O_3@BPO_4 sandwich-like hollow spheres as metal-free supported liquid-phase catalysts. *J. Catal.* **381**, 599-607 (2020).

21 Venegas, J. M. *et al.* Why Boron Nitride is such a Selective Catalyst for the Oxidative

Dehydrogenation of Propane. *Angew. Chem. Int. Ed.* **59**, 16527-16535 (2020).

22 Liu, Z., Lu, W.-D., Wang, D. & Lu, A.-H. Interplay of On- and Off-Surface Processes in the B_2O_3-Catalyzed Oxidative Dehydrogenation of Propane: A DFT Study. *J. Phys. Chem. C* **125**, 24930-24944 (2021).
